# Rhythms in Remodeling: Posttranslational Regulation of Bone by the Circadian Clock

**DOI:** 10.3390/biomedicines13030705

**Published:** 2025-03-13

**Authors:** Vincent G. Yuan

**Affiliations:** Department of Otolaryngology–Head & Neck Surgery, University of Pittsburgh, Pittsburgh, PA 15213, USA; vincentyuan@pitt.edu

**Keywords:** circadian clock, bone remodeling, osteoblasts, osteoclasts, osteocytes, posttranslational modifications (PTMs), phosphorylation, acetylation, ubiquitination, skeletal homeostasis, osteoporosis, circadian rhythm disruptions, bone metabolism

## Abstract

The circadian clock is a fundamental timekeeping system that regulates rhythmic biological processes in response to environmental light–dark cycles. In mammals, core clock genes (CLOCK, BMAL1, PER, and CRY) orchestrate these rhythms through transcriptional–translational feedback loops, influencing various physiological functions, including bone remodeling. Bone homeostasis relies on the coordinated activities of osteoblasts, osteoclasts, and osteocytes, with increasing evidence highlighting the role of circadian regulation in maintaining skeletal integrity. Disruptions in circadian rhythms are linked to bone disorders such as osteoporosis. Posttranslational modifications (PTMs), including phosphorylation, acetylation, and ubiquitination, serve as crucial regulators of both circadian mechanisms and bone metabolism. However, the specific role of PTMs in integrating circadian timing with bone remodeling remains underexplored. This review examines the intersection of circadian regulation and PTMs in bone biology, elucidating their impact on bone cell function and homeostasis. Understanding these interactions may uncover novel therapeutic targets for skeletal diseases associated with circadian disruptions.

## 1. Introduction

The circadian clock, an intrinsic timekeeping system, orchestrates rhythmic biological processes in virtually all organisms, aligning physiological functions with environmental cycles of light and dark. In mammals, a group of core clock genes—CLOCK, BMAL1, PER, and CRY—regulate this system through transcriptional–translational feedback loops [1,2,3,4]. Beyond governing daily physiological processes like rest–activity patterns and energy balance, circadian cycles significantly impact tissue-specific functions, such as bone remodeling [5,6,7].

**Figure 1:**
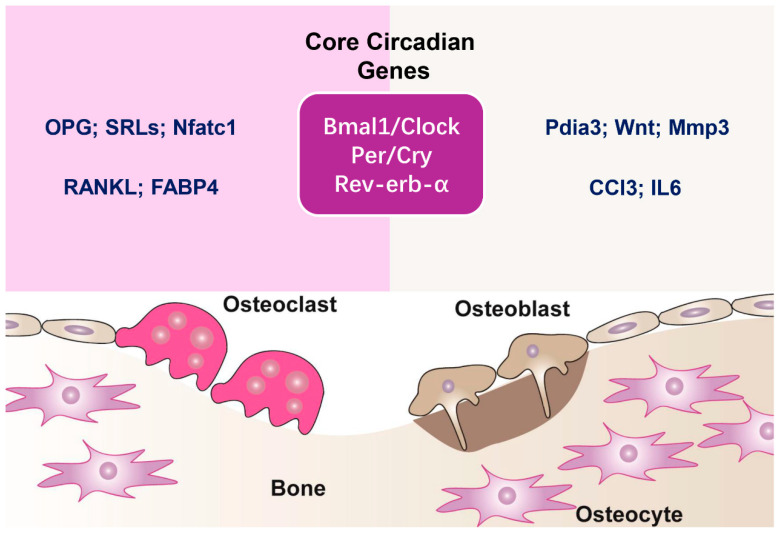
Circadian regulation of bone remodeling. Bone remodeling is a continuous process driven by the balanced activity of osteoclasts, responsible for bone resorption, and osteoblasts, which facilitate new bone formation. Maintaining this equilibrium is crucial for preserving skeletal health. Osteoclasts degrade the bone matrix, releasing minerals into circulation, while osteoblasts synthesize and mineralize new bone tissue. Core genes of the circadian clock are fundamental in controlling bone remodeling. The Bmal1/Clock complex initiates the expression of circadian-regulated genes, while Per/Cry and Rev-erb-α function as suppressors, creating rhythmic gene expression patterns that impact osteoclast and osteoblast activity. In osteoclasts, circadian genes influence bone resorption by controlling essential regulators, including osteoprotegerin (OPG), sclerostin (SRTs), Nfatc1, RANKL, and FABP4. In osteoblasts, circadian control influences bone formation through pathways involving protein disulfide isomerase family A member 3 (Pdia3), Wnt signaling, matrix metalloproteinase-3 (Mmp3), chemokine (C-C motif) ligand 3 (CCl3), and interleukin-6 (IL6). These molecular interactions underscore the critical influence of circadian rhythms in maintaining skeletal balance.

Bone is a constantly evolving tissue that maintains its structure through the coordinated actions of osteoblasts, osteoclasts, and osteocytes [8,9,10,11]. Growing research indicates that the circadian clock plays a crucial role in regulating bone cell activity and maintaining bone homeostasis, while disturbances in circadian rhythms are associated with bone disorders like osteoporosis [12,13,14,15]. Peripheral clocks in bone cells function alongside the central clock, translating time-related signals into localized activities, including bone development, resorption, and mineralization (Figure 1) [12,16,17].

Posttranslational modifications (PTMs) are essential for precisely regulating circadian timing mechanisms and influencing downstream cellular functions [18,19,20]. PTMs, such as phosphorylation, acetylation, and ubiquitination, function as key regulators that influence the stability, distribution, and activity of proteins essential for circadian regulation and bone metabolism [21,22,23,24,25,26]. Despite the growing understanding of circadian regulation in bone biology, the role of PTMs in linking these two systems remains underexplored.

This review seeks to offer an in-depth analysis of the role of biological timing mechanisms in regulating bone health through PTMs. We discuss the mechanisms of PTMs in clock protein regulation, their impact on bone cell function, and the potential implications for circadian rhythm disruptions on skeletal integrity. Understanding these interactions may reveal novel therapeutic avenues for bone-related diseases.

## 2. Circadian Clock Mechanisms in Bone

The biological clock operates through a network of central and peripheral timekeepers that regulate both cellular and systemic rhythms [27]. The central clock, located in the suprachiasmatic nucleus (SCN) of the hypothalamus, synchronizes peripheral clocks across various tissues, including bone, by responding to environmental light cues [12,28].

At the molecular level, the circadian mechanism relies on a transcriptional–translational feedback loop involving CLOCK/BMAL1, PER1/PER2, and CRY1/CRY2 [29]. CLOCK and BMAL1 form a heterodimer that activates the transcription of *Per* and *Cry* genes. Once translated, PER and CRY proteins accumulate in the cytoplasm, dimerize, and translocate into the nucleus, where they inhibit CLOCK/BMAL1 activity, thereby completing the feedback loop [29].

In bone, the circadian clock regulates the function of bone-forming, resorbing, and maintaining cells, orchestrating the remodeling process in a time-dependent manner [12,30]. BMAL1 and CLOCK regulate osteoblast differentiation and bone formation, as their deletion reduces bone mass [13,31,32]. BMAL1 also influences osteoclasts, where its loss leads to increased bone mass due to reduced resorption [17]. PER1/2 knockout mice show higher bone mass, indicating their role in bone formation. Similarly, CRY1/2 deficiency enhances osteoblast activity and bone formation (Table 1) [33].

Recent studies highlight the interaction between systemic and local timing mechanisms in maintaining bone health. Circadian rhythm disruptions, including those caused by irregular work schedules or prolonged travel across time zones, disturb the synchronization of bone remodeling, elevating the risk of osteoporosis and fractures [34]. Additionally, hormonal regulators like glucocorticoids and parathyroid hormone, both of which follow circadian patterns, further integrate systemic rhythms with local bone clocks [14,35,36].

Understanding the mechanisms by which circadian clocks govern bone biology offers valuable insights into skeletal physiology and pathology. These findings lay the groundwork for exploring therapeutic strategies that target circadian rhythms to treat bone-related diseases, such as utilizing melatonin agonists or chronotherapy approaches to mitigate bone loss and improve skeletal health.

## 3. Overview of Posttranslational Modifications

PTMs are chemical changes that proteins undergo after synthesis, playing a crucial role in regulating their structure, stability, localization, and function [37,38,39,40]. These modifications allow for rapid and reversible changes, enabling proteins to respond dynamically to cellular and environmental cues [41,42]. Within the circadian clock system, PTMs play a vital role in ensuring rhythmic accuracy and stability, regulating both clock protein function and associated downstream pathways [18].

**Figure 2:**
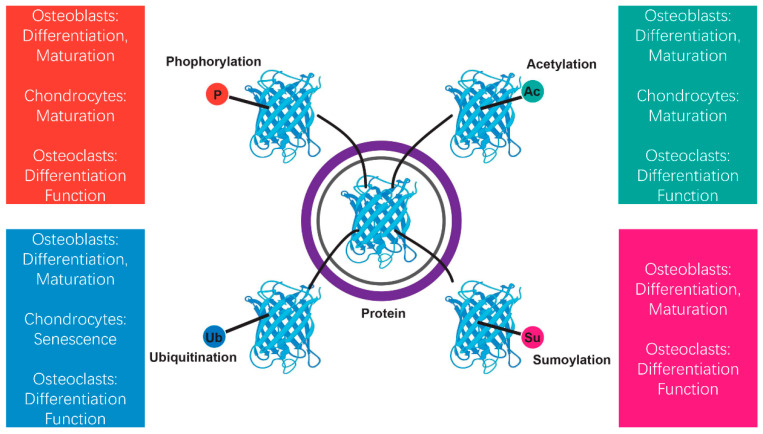
Regulation of bone remodeling through protein modifications. Bone remodeling is a highly controlled process that relies on the coordinated actions of osteoblasts, chondrocytes, and osteoclasts to regulate bone formation, cartilage maturation, and resorption. Protein modifications after translation are essential for regulating the stability, activity, and function of key signaling molecules throughout this process. This study focuses on four major protein modifications—phosphorylation, acetylation, ubiquitination, and sumoylation—in bone remodeling. These PTMs collectively orchestrate the cellular processes necessary for maintaining bone homeostasis and skeletal integrity.

Phosphorylation, acetylation, ubiquitination, and sumoylation are among the most prevalent posttranslational modifications, each of which plays distinct roles in regulating circadian biology and bone metabolism (Figure 2) [21,22,43,44]. Phosphorylation, regulated by kinases like casein kinase 1 (CK1), influences the durability and transport of key circadian proteins such as PER within the nucleus [45]. Acetylation and deacetylation, controlled by enzymes such as CLOCK and SIRT1, influence chromatin remodeling and gene expression, linking circadian rhythms to metabolic and skeletal processes [46,47]. Ubiquitination, facilitated by E3 ligases, regulates circadian protein breakdown via the proteasome pathway, maintaining appropriate turnover and enabling clock phase adjustments [48]. Sumoylation, though less studied in circadian systems, has been implicated in regulating transcriptional activity and protein interactions, with potential impacts on bone homeostasis [49].

**Figure 3:**
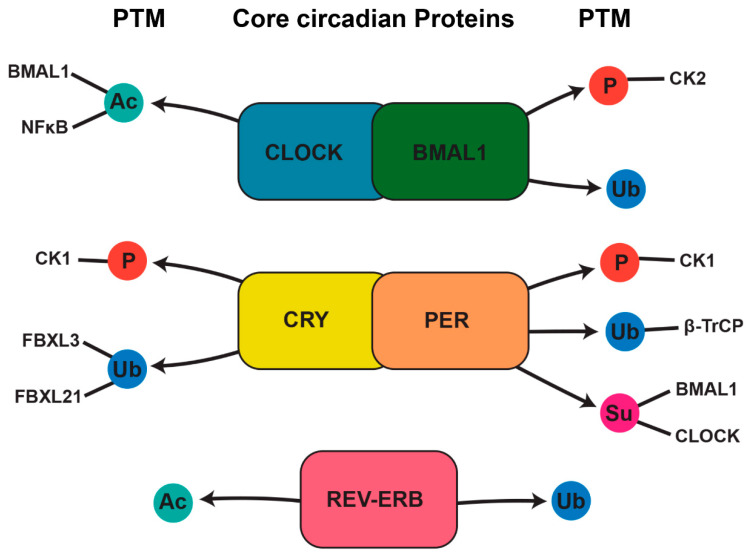
Circadian regulation of protein modifications in bone remodeling. Core circadian proteins, including CLOCK/BMAL1, CRY/PER, and REV-ERB, play essential roles in regulating posttranslational modifications that influence bone remodeling. These proteins regulate rhythmic variations in phosphorylation, acetylation, sumoylation, and ubiquitination, influencing the stability and function of critical signaling molecules responsible for bone formation and resorption. CLOCK regulates the acetylation of BMAL1 and NF-κB, impacting transcriptional activity in bone cells. BMAL1, in turn, controls the phosphorylation of casein kinase 2 (CK2), a key regulator of osteoblast function, while BMAL1 ubiquitination also contributes to bone remodeling by modulating protein stability and degradation. CRY and PER influence phosphorylation and protein interactions critical for circadian regulation. CRY controls the phosphorylation of casein kinase 1 (CK1) and interacts with ubiquitin ligases FBXL3 and FBXL21, which regulate protein degradation. Similarly, PER regulates CK1 phosphorylation and the sumoylation of BMAL1/CLOCK, affecting transcriptional feedback loops. Additionally, PER interacts with β-TrCP, a component of the ubiquitin–proteasome system. REV-ERB further integrates the circadian control of protein modifications by associating with acetylation- and ubiquitination-related proteins, influencing bone homeostasis.

These PTMs not only regulate circadian clock components but also intersect with signaling pathways involved in bone remodeling (Figure 3). For example, the phosphorylation of RANKL signaling components in osteoclasts and the acetylation of transcription factors in osteoblasts highlight the functional overlap between circadian PTMs and bone cell activity [12,50].

Understanding these modifications provides a mechanistic basis for how circadian rhythms and bone health are interconnected, with potential therapeutic implications, including the development of kinase inhibitors targeting aberrant phosphorylation or small molecules modulating acetylation to enhance bone regeneration.

## 4. Posttranslational Regulation of Bone Through the Circadian Timing System

### 4.1. Phosphorylation’s Role in Circadian Rhythms and Bone Regulation

Phosphorylation is a key posttranslational modification, carried out by kinases, that actively controls protein activity, location, and stability [51]. In circadian systems, phosphorylation is crucial for sustaining rhythmicity, and its effects reach bone biology by regulating the functions of osteoclasts and osteoblasts [12].

Casein kinase 1 (CK1) is a central kinase in the circadian system, responsible for phosphorylating PER proteins, which are essential elements of the circadian negative feedback loop [45,52]. CK1-mediated phosphorylation regulates the stability and nuclear positioning of PER proteins, thereby controlling the overall length of the circadian cycle [53]. In bone cells, CK1 is involved in regulating processes like differentiation and mineralization [54]. Another crucial kinase, AMP-activated protein kinase (AMPK), plays a role in metabolic regulation and connects energy balance to circadian rhythms [55]. AMPK phosphorylates and destabilizes CRY proteins, resetting the clock in response to energy status [55,56]. In bone, AMPK influences osteoclastogenesis and osteoblast function, providing a metabolic–circadian interface that is critical for skeletal health [57].

In osteoclasts, phosphorylation events downstream of circadian clock components influence bone resorption [12]. For example, RANKL signaling, which drives osteoclast differentiation and activity, exhibits circadian oscillations, with the phosphorylation-dependent regulation of NF-κB and other downstream targets [58,59]. This rhythmic control ensures time-of-day-specific bone resorption, aligning with systemic circadian cues [12]. Osteoblasts, responsible for bone formation, also exhibit circadian-regulated phosphorylation dynamics [14]. BMAL1, a key clock protein, interacts with signaling pathways like Wnt/β-catenin, which are regulated by phosphorylation, to control osteoblast differentiation and function [60,61,62,63,64]. The suppression of Bmal1 in osteoblasts reduced GSK-3β phosphorylation at serine 9, thereby impacting osteoblast differentiation [63]. Interestingly, the deletion of Bmal1 in osteoblasts increased Bmp2 expression and SMAD1 phosphorylation, resulting in enhanced osteoblast differentiation and activity [65]. Additionally, the phosphorylation of RUNX2, an essential transcription factor for osteogenesis, is controlled by circadian-regulated kinases, influencing its stability and activity [66,67].

### 4.2. Acetylation and Deacetylation in Circadian and Bone Regulation

Acetylation and deacetylation are key posttranslational modifications that regulate protein function and gene expression [68]. In circadian biology, these processes are crucial for the transcriptional and epigenetic regulation of clock components, while in bone metabolism, they play a role in the differentiation and activity of osteoclasts and osteoblasts. Circadian clock genes present promising therapeutic targets for addressing bone loss [69,70].

Histone acetyltransferases (HATs), such as CLOCK and p300/CBP, are critical for circadian regulation and bone metabolism [71,72]. CLOCK, a core circadian transcription factor, possesses intrinsic HAT activity that acetylates histone H3 at lysine 14 (H3K14), promoting the transcription of clock-controlled genes [71]. Similarly, p300/CBP-mediated acetylation activates osteoblast-specific transcription factors like RUNX2, enhancing osteoblast differentiation and bone formation [73].Conversely, sirtuins, particularly SIRT1, serve as deacetylases that counterbalance the activity of HATs [74]. SIRT1 deacetylates key circadian proteins, including PER2 and BMAL1, modulating their stability and transcriptional activity to sustain circadian oscillations and bone homeostasis [75,76,77].

The circadian clock regulates the rhythmic acetylation and deacetylation of both histone and non-histone proteins, coordinating bone remodeling processes with systemic circadian signals [78]. The acetylation of histones at promoter regions of bone-specific genes follows circadian oscillations, driven by HATs such as CLOCK and deacetylation mediated by SIRT1 [47,79]. These rhythmic modifications affect the transcription of genes involved in osteoblast activity, including ALP and COL1A1, as well as genes related to osteoclast differentiation, such as RANKL and OPG [47,80,81,82,83].

Studies have demonstrated that disruptions in circadian acetylation pathways impair bone remodeling. For instance, the deletion of SIRT1 in osteoblasts leads to defective bone formation and delayed fracture healing, underscoring its role in coordinating circadian rhythms with skeletal homeostasis [84]. Similarly, the disrupted acetylation of RUNX2 due to circadian disturbances affects osteoblast differentiation and bone mineralization [67].

### 4.3. Ubiquitination in Circadian and Bone Regulation

Ubiquitination is an essential posttranslational modification that controls protein degradation, stability, and cellular localization, impacting a broad array of biological processes [85]. In the context of circadian rhythms and bone metabolism, ubiquitination plays a crucial role in adjusting the timing and amplitude of circadian clock components, as well as maintaining bone homeostasis by regulating osteoblast and osteoclast activity [86].

The ubiquitin–proteasome system (UPS) is vital for regulating circadian rhythms by managing the turnover of clock proteins [87]. E3 ubiquitin ligases, such as β-TrCP and FBXL3, facilitate the ubiquitination of core clock proteins, including PER1, PER2, and CRY, which are essential regulators of the circadian clock’s negative feedback loop [88]. For instance, β-TrCP promotes the degradation of PER proteins through ubiquitination, whereas FBXL3 targets CRY proteins for degradation via the proteasome [88,89,90,91]. These ubiquitination processes control the duration of the circadian cycle and maintain proper rhythmicity [92]. In bone cells, these ubiquitin ligases also influence the circadian control of gene expression, contributing to bone remodeling processes.

In osteoblasts, ubiquitination affects the regulation of transcription factors like RUNX2 and Osterix [93]. RUNX2, a key regulator of osteoblast differentiation and bone formation, is degraded by the ubiquitin–proteasome system to regulate its activity [94]. For instance, the E3 ligase SMURF1 mediates the ubiquitination of RUNX2, thereby limiting its transcriptional activity [95]. The disruption of this pathway can lead to abnormal osteoblast differentiation and excessive bone formation, which may contribute to skeletal diseases like osteosclerosis [96].

Similarly, the differentiation and function of osteoclasts, which are responsible for bone resorption, are tightly regulated by ubiquitination [97]. NF-κB, a key signaling molecule involved in osteoclastogenesis, is regulated by the E3 ligase TRAF6. TRAF6 catalyzes the polyubiquitination of various signaling proteins in the RANKL pathway, activating NF-κB and promoting osteoclast differentiation [98]. Furthermore, deubiquitinating enzymes (DUBs) such as CYLD modulate the activity of NF-κB by removing polyubiquitin chains, ensuring the proper regulation of bone resorption [99]. The circadian regulation of these ubiquitination processes helps synchronize bone remodeling with the body’s daily rhythm.

The circadian control of ubiquitination in bone cells ensures a balanced rhythm between bone formation and resorption. For example, the E3 ligase FBXL3, known for controlling circadian rhythms, also influences osteoblast activity by regulating the stability of key transcription factors like RUNX2 [100]. Rhythmic oscillations in the expression of ubiquitin ligases and deubiquitinating enzymes help coordinate the timing of osteoclastogenesis and osteoblast differentiation, aligning bone turnover with systemic circadian cues [101].

Disruptions in the ubiquitination machinery, such as mutations in E3 ligases or DUBs, can lead to dysregulated circadian rhythms, which in turn affect bone homeostasis [48]. Studies have shown that circadian disruption via altered expression of ubiquitin ligases can result in impaired bone turnover, with a preference for either excessive bone resorption or formation [102,103]. These findings emphasize the importance of maintaining proper circadian regulation of the ubiquitin–proteasome system for skeletal health.

### 4.4. Sumoylation in Circadian and Bone Regulation

Sumoylation is a posttranslational modification where a small ubiquitin-like modifier (SUMO) protein is covalently attached to target proteins. This modification is crucial for regulating protein stability, localization, and activity, and it has been linked to the regulation of various cellular processes, including circadian rhythms and bone metabolism [37,104]. Sumoylation is a highly regulated process, with specific enzymes responsible for attaching and removing SUMO moieties. This modification affects the activity of a broad range of proteins, including transcription factors and clock-related genes, and can influence bone remodeling by modulating the balance between osteoblast and osteoclast activity [24,105,106].

Sumoylation is essential for regulating circadian rhythms by influencing the activity of core clock proteins like BMAL1 and CLOCK [104]. A well-established role of sumoylation in circadian regulation is its impact on transcriptional regulators. Sumoylation modifies BMAL1, a core element of the circadian activator complex, affecting its interactions with proteins like CLOCK and its ability to regulate circadian gene expression [107]. In living organisms, BMAL1 undergoes sumoylation at the highly conserved Lys259 residue. This modification is crucial for maintaining its circadian expression and overall clock rhythmicity, as shown by studies involving the expression of a BMAL1 variant lacking SUMO attachment [108].

Likewise, PER proteins, essential for the circadian clock’s negative feedback mechanism, also undergo sumoylation. This modification enhances their stability and interactions with other clock components, ultimately affecting the duration of the circadian cycle [24]. These observations indicate that sumoylation plays a crucial role in refining circadian rhythms by modulating the stability and interactions of clock-related proteins.

In bone metabolism, sumoylation influences essential regulators that control osteoblast and osteoclast differentiation and activity. RUNX2, a critical transcription factor for osteoblast differentiation, is known to be modulated by sumoylation, which influences its ability to regulate bone matrix formation and mineralization [109]. Sumoylation stabilizes RUNX2 and boosts its transcriptional function, facilitating osteoblast differentiation and bone development [49]. Additionally, sumoylation modifies transcription factors like Osterix, playing a role in regulating osteoblast function and impacting bone formation [110].

Sumoylation plays a role in osteoclastogenesis by influencing the transition of precursor cells into mature osteoclasts. It regulates NFATc1, a key transcription factor that is essential for osteoclast development [111]. The regulation of NFATc1 has been shown to enhance its transcriptional activity and promote osteoclast differentiation, which is important for bone resorption [112]. Furthermore, the sumoylation of other signaling molecules, such as the transcription factor C/EBPβ, has been implicated in regulating osteoclastogenesis by modulating gene expression involved in bone resorption [113,114].

### 4.5. Links Between Posttranslational Modifications Induced by Circadian Clock Dysfunction and Bone Disorders

**Figure 4:**
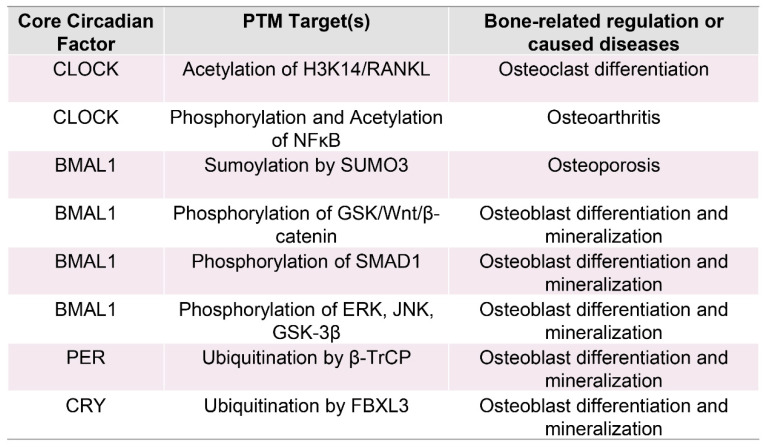
Core circadian factors, posttranslational modifications, and their roles in bone remodeling. This table presents the key circadian regulators—CLOCK, BMAL1, PER, and CRY—and their interactions with PTM-associated proteins. These alterations are essential for controlling bone remodeling and have been linked to various bone disorders. CLOCK promotes osteoclast differentiation through the acetylation of H3K14 and regulation of RANKL and contributes to osteoarthritis via phosphorylation and acetylation of NF-κB. BMAL1 regulates bone formation through various mechanisms, such as SUMO3-mediated sumoylation (associated with reduced bone mass) and phosphorylation processes that impact GSK/Wnt/β-catenin, SMAD1, ERK, JNK, and GSK-3β, all of which influence osteoblast development and bone mineralization. PER contributes to osteoblast development and mineralization through β-TrCP-mediated ubiquitination, whereas CRY influences these processes via FBXL3-driven ubiquitination. These findings underscore the complex interplay between circadian regulation, PTMs, and skeletal homeostasis, highlighting potential therapeutic targets for bone-related disorders.

Circadian rhythm disruptions, whether due to shift work, sleep disturbances, or genetic alterations in clock genes, can result in the improper regulation of posttranslational modifications (PTMs) [115,116]. This imbalance has been linked to disruptions in bone remodeling, contributing to conditions such as osteoporosis, metabolic bone diseases, and inflammatory bone disorders (Figure 4) [26,44,117]. Understanding how circadian clock dysfunction impacts PTMs and, subsequently, bone health is critical for identifying novel therapeutic approaches for these bone-related conditions.

In genetic bone disorders such as osteogenesis imperfecta, achondroplasia, and Marfan syndrome, mutations in structural proteins like collagen or growth factors disrupt bone integrity [118,119]. While circadian dysfunction is not directly responsible for these disorders, it may impact PTMs critical to bone health, such as the phosphorylation of growth factors or collagen enzymes. In osteogenesis imperfecta, for example, the circadian-controlled posttranslational regulation of collagen production could potentially modulate bone fragility [120,121]. Moreover, in achondroplasia, where cartilage growth is impaired due to mutations in the FGFR3 gene, the circadian regulation of signaling pathways like MAPK may affect posttranslational modifications of proteins involved in bone growth, potentially exacerbating disease severity [122,123].

Posttranslational regulation influenced by the circadian clock also plays a role in metabolic bone disorders, including osteoporosis, rickets, osteomalacia, and Paget’s disease [116,124]. In osteoporosis, posttranslational modifications such as the phosphorylation of proteins involved in bone resorption (e.g., RANKL and NF-κB) are regulated by circadian rhythms [32,47,125]. Disruptions in phosphorylation can interfere with the equilibrium of osteoblast and osteoclast activity, resulting in bone deterioration [126]. Similarly, in rickets and osteomalacia, where vitamin D metabolism is impaired, circadian disruption affects PTMs of vitamin D receptors and enzymes critical for calcium homeostasis, exacerbating bone mineralization defects [127,128]. In Paget’s disease, which involves irregular bone turnover, the circadian control of PTMs regulating osteoclast development and activity contributes to disease advancement [125,129].

The posttranslational control of inflammatory cytokines, such as TNF-α, IL-1β, and IL-6, is crucial in autoimmune bone disorders like rheumatoid arthritis and ankylosing spondylitis [130]. The circadian clock governs the production and activation of these cytokines, and their posttranslational modifications (such as acetylation or phosphorylation) influence their ability to promote bone erosion and inflammation [131]. The dysregulation of these PTMs can worsen inflammatory bone diseases, and therapies targeting circadian rhythms may help modulate these processes, potentially leading to improved outcomes in RA and AS [21,116].

In bone cancers such as osteosarcoma, Ewing sarcoma, and multiple myeloma, circadian disruption can impact posttranslational modifications of proteins that regulate the cell cycle and suppress tumor growth [22,132]. The dysregulated phosphorylation of tumor suppressors such as p53 or Rb may lead to unrestrained cell proliferation and tumor progression [133]. Research suggests that targeting circadian rhythms to optimize the timing of chemotherapy could improve treatment efficacy by aligning therapeutic windows with the body’s natural rhythms of cell cycle regulation [134]. Exploring the impact of circadian clock-regulated PTMs on tumor progression and bone metastasis may pave the way for more targeted and personalized treatments for individuals with bone cancer.

### 4.6. Future Directions and Clinical Implications

While significant progress has been made in understanding how circadian rhythms and PTMs regulate bone metabolism, key gaps remain. The precise timing and dynamics of PTMs—such as phosphorylation, acetylation, and ubiquitination—in osteoblast and osteoclast activity are not fully understood. Additionally, the impact of circadian disruption (e.g., shift work, jet lag) on PTM-mediated bone remodeling requires further investigation [34]. Recent advancements in high-resolution omics technologies provide powerful tools to address these challenges.

Time-resolved phosphoproteomics enables the mapping of circadian fluctuations in phosphorylation patterns, revealing how kinase and phosphatase activities regulate bone cell function across the day–night cycle [135]. Single-cell multiomics, integrating transcriptomics and proteomics, allows for the cell-type-specific analysis of PTMs, elucidating how osteoblasts, osteoclasts, and osteocytes differentially respond to circadian cues [136]. Additionally, CRISPR-based genome editing and epigenetic profiling can help dissect how PTM-modified clock proteins contribute to bone homeostasis, identifying potential therapeutic targets [137]. The real-time imaging of PTMs using fluorescence-based biosensors provides dynamic insights into PTM regulation within living bone tissue, deepening our understanding of the molecular interplay between circadian rhythms and skeletal remodeling [138].

Incorporating circadian biology into osteoporosis treatment could enhance therapeutic outcomes. Since bone turnover follows a daily rhythm, optimizing drug timing—such as bisphosphonate administration—may improve efficacy while minimizing side effects. Similarly, time-specific recommendations for weight-bearing exercise and calcium/vitamin D intake could better align with peak bone metabolism [139]. For individuals with circadian disruptions, tailored interventions to restore rhythmic bone remodeling may help mitigate bone loss.

PTM-targeted therapies may also benefit from circadian considerations. Since modifications like phosphorylation and acetylation fluctuate throughout the day, aligning treatments with these cycles could optimize bone formation and resorption dynamics. Biomarker-driven omics approaches could further enable the prediction of individual PTM and circadian profiles, paving the way for precision medicine in bone disease management [140]. A deeper understanding of these interactions may lead to personalized, time-sensitive interventions for osteoporosis and fracture healing. As research advances, integrating circadian principles into clinical guidelines could drive more effective, individualized strategies for maintaining skeletal health.

## 5. Conclusions

Circadian rhythms play a pivotal role in bone metabolism by regulating PTMs that influence osteoclast and osteoblast activity. Disruptions in these rhythms are increasingly linked to bone disorders such as osteoporosis. Future research should focus on unraveling the precise mechanisms of circadian–PTM interactions and developing chronotherapy-based interventions. Understanding the temporal dynamics of bone biology could lead to optimized treatments for skeletal disorders, enhancing long-term bone health.

## Figures and Tables

**Table 1:** Circadian clock components and their roles in bone homeostasis in mice.

Molecular Player	Target Bone Cells	Effects on Bone Metabolism	Experimental Evidence
BMAL1/CLOCK	Osteoblasts	Regulates osteoblast differentiation and bone formation	Osteoblast-specific Bmal1 knockout mice exhibit reduced bone mass due to impaired osteoblast differentiation. CLOCK-mutation mice show decreased bone formation, suggesting its role in osteoblast function.
	Osteoclasts	Influences osteoclast differentiation and bone resorption	Osteoclast-specific Bmal1 knockout mice display high bone mass due to reduced osteoclast differentiation.
PER1/PER2	Osteoblasts	Regulates osteoblast proliferation and bone formation	Per1/Per2 knockout mice exhibit increased bone mass.
CRY1/CRY2	Osteoblasts	Modulates osteoblast proliferation and bone formation	Cry1/Cry2 double knockout mice show increased bone formation and osteoblast activity.

## Abbreviations

The following abbreviations are used in this manuscript:

CLOCK	Circadian locomotor output cycles kaput
BMAL1	Basic helix-loop-helix ARNT-like protein 1
PER	Period
CRY	Cryptochrome
PTMs	Posttranslational modifications
SCN	Suprachiasmatic nucleus
RANKL	Receptor activator of nuclear factor kappa-B ligand
CK1	Casein kinase 1
SIRT1	Sirtuin 1
AMPK	AMP-activated protein kinase
NF-κB	Nuclear factor kappa B subunit 1
SMAD1	Mothers against decapentaplegic homolog 1
RUNX2	Runt-related transcription factor 2
GSK-3β	Glycogen synthase kinase 3 beta
p300/CBP	CREBBP
HATs	Histone acetyltransferases
ALP	Alkaline phosphatases
COL1A1	Collagen, type I
OPG	Osteoprotegerin
UPS	Ubiquitin–proteasome system
β-TrCP	beta-transducin repeat-containing protein
FBXL3	F-box and leucine rich repeat protein 3
SMURF1	Smad ubiquitination regulation factor 1
TRAF6	TNF receptor-associated factor 6
DUBs	Deubiquitinating enzymes
CYLD	Cylindromatosis
NFATc1	TF nuclear factor of activated T cells 1
C/EBPβ	CCAAT/enhancer-binding protein beta
FGFR3	Fibroblast growth factor receptor 3
MAPK	Mitogen activated protein kinase
RA	Rheumatoid arthritis
AS	Ankylosing spondylitis

## References

[1] Chaput J.P., McHill A.W., Cox R.C., Broussard J.L., Dutil C., da Costa B.G.G., Sampasa-Kanyinga H., Wright K.P. (2023). The role of insufficient sleep and circadian misalignment in obesity. Nat. Rev. Endocrinol..

[2] Patke A., Young M.W., Axelrod S. (2020). Molecular mechanisms and physiological importance of circadian rhythms. Nat. Rev. Mol. Cell Biol..

[3] Ayyar V.S., Sukumaran S. (2021). Circadian rhythms: Influence on physiology, pharmacology, and therapeutic interventions. J. Pharmacokinet. Pharmacodyn..

[4] Fagiani F., Di Marino D., Romagnoli A., Travelli C., Voltan D., Di Cesare Mannelli L., Racchi M., Govoni S., Lanni C. (2022). Molecular regulations of circadian rhythm and implications for physiology and diseases. Signal Transduct. Target. Ther..

[5] Bouvard B., Mabilleau G. (2024). Gut hormones and bone homeostasis: Potential therapeutic implications. Nat. Rev. Endocrinol..

[6] Kawai M., Rosen C.J. (2010). PPARgamma: A circadian transcription factor in adipogenesis and osteogenesis. Nat. Rev. Endocrinol..

[7] de Crombrugghe B. (2005). Osteoblasts clock in for their day job. Cell.

[8] Raggatt L.J., Partridge N.C. (2010). Cellular and molecular mechanisms of bone remodeling. J. Biol. Chem..

[9] Dirckx N., Moorer M.C., Clemens T.L., Riddle R.C. (2019). The role of osteoblasts in energy homeostasis. Nat. Rev. Endocrinol..

[10] Salazar V.S., Gamer L.W., Rosen V. (2016). BMP signalling in skeletal development, disease and repair. Nat. Rev. Endocrinol..

[11] Chotiyarnwong P., McCloskey E.V. (2020). Pathogenesis of glucocorticoid-induced osteoporosis and options for treatment. Nat. Rev. Endocrinol..

[12] Luo B., Zhou X., Tang Q., Yin Y., Feng G., Li S., Chen L. (2021). Circadian rhythms affect bone reconstruction by regulating bone energy metabolism. J. Transl. Med..

[13] Yuan G., Hua B., Yang Y., Xu L., Cai T., Sun N., Yan Z., Lu C., Qian R. (2017). The Circadian Gene Clock Regulates Bone Formation Via PDIA3. J. Bone Miner. Res..

[14] Kikyo N. (2024). Circadian Regulation of Bone Remodeling. Int. J. Mol. Sci..

[15] Yang N., Meng Q.J. (2016). Circadian Clocks in Articular Cartilage and Bone: A Compass in the Sea of Matrices. J. Biol. Rhythms.

[16] Richards J., Gumz M.L. (2012). Advances in understanding the peripheral circadian clocks. FASEB J..

[17] Xu C., Ochi H., Fukuda T., Sato S., Sunamura S., Takarada T., Hinoi E., Okawa A., Takeda S. (2016). Circadian Clock Regulates Bone Resorption in Mice. J. Bone Miner. Res..

[18] Mehra A., Baker C.L., Loros J.J., Dunlap J.C. (2009). Post-translational modifications in circadian rhythms. Trends Biochem. Sci..

[19] Yan J., Kim Y.J., Somers D.E. (2021). Post-Translational Mechanisms of Plant Circadian Regulation. Genes.

[20] Li W., Li F., Zhang X., Lin H.K., Xu C. (2021). Insights into the post-translational modification and its emerging role in shaping the tumor microenvironment. Signal Transduct. Target. Ther..

[21] Wu X., Xu M., Geng M., Chen S., Little P.J., Xu S., Weng J. (2023). Targeting protein modifications in metabolic diseases: Molecular mechanisms and targeted therapies. Signal Transduct. Target. Ther..

[22] Chen L., Liu S., Tao Y. (2020). Regulating tumor suppressor genes: Post-translational modifications. Signal Transduct. Target. Ther..

[23] Duszka K., Wahli W. (2020). Peroxisome Proliferator-Activated Receptors as Molecular Links Between Caloric Restriction and Circadian Rhythm. Nutrients.

[24] Chen L.C., Hsieh Y.L., Tan G.Y.T., Kuo T.Y., Chou Y.C., Hsu P.H., Hwang-Verslues W.W. (2021). Differential effects of SUMO1 and SUMO2 on circadian protein PER2 stability and function. Sci. Rep..

[25] Stojkovic K., Wing S.S., Cermakian N. (2014). A central role for ubiquitination within a circadian clock protein modification code. Front. Mol. Neurosci..

[26] Stechschulte L.A., Czernik P.J., Rotter Z.C., Tausif F.N., Corzo C.A., Marciano D.P., Asteian A., Zheng J., Bruning J.B., Kamenecka T.M. (2016). PPARG Post-translational Modifications Regulate Bone Formation and Bone Resorption. EBioMedicine.

[27] de Assis L.V.M., Oster H. (2021). The circadian clock and metabolic homeostasis: Entangled networks. Cell Mol. Life Sci..

[28] Astiz M., Heyde I., Oster H. (2019). Mechanisms of Communication in the Mammalian Circadian Timing System. Int. J. Mol. Sci..

[29] Takahashi J.S. (2017). Transcriptional architecture of the mammalian circadian clock. Nat. Rev. Genet..

[30] Morris H., Goncalves C.F., Dudek M., Hoyland J., Meng Q.J. (2021). Tissue physiology revolving around the clock: Circadian rhythms as exemplified by the intervertebral disc. Ann. Rheum. Dis..

[31] Tsang K., Liu H., Yang Y., Charles J.F., Ermann J. (2019). Defective circadian control in mesenchymal cells reduces adult bone mass in mice by promoting osteoclast function. Bone.

[32] Takarada T., Xu C., Ochi H., Nakazato R., Yamada D., Nakamura S., Kodama A., Shimba S., Mieda M., Fukasawa K. (2017). Bone Resorption Is Regulated by Circadian Clock in Osteoblasts. J. Bone Miner. Res..

[33] Fu L., Patel M.S., Bradley A., Wagner E.F., Karsenty G. (2005). The molecular clock mediates leptin-regulated bone formation. Cell.

[34] Swanson C.M., Kohrt W.M., Buxton O.M., Everson C.A., Wright K.P., Orwoll E.S., Shea S.A. (2018). The importance of the circadian system & sleep for bone health. Metabolism.

[35] Fujihara Y., Kondo H., Noguchi T., Togari A. (2014). Glucocorticoids mediate circadian timing in peripheral osteoclasts resulting in the circadian expression rhythm of osteoclast-related genes. Bone.

[36] Silva B.C., Bilezikian J.P. (2015). Parathyroid hormone: Anabolic and catabolic actions on the skeleton. Curr. Opin. Pharmacol..

[37] Lee J.M., Hammaren H.M., Savitski M.M., Baek S.H. (2023). Control of protein stability by post-translational modifications. Nat. Commun..

[38] Wang Y.C., Peterson S.E., Loring J.F. (2014). Protein post-translational modifications and regulation of pluripotency in human stem cells. Cell Res..

[39] Beltrao P., Bork P., Krogan N.J., van Noort V. (2013). Evolution and functional cross-talk of protein post-translational modifications. Mol. Syst. Biol..

[40] Tootle T.L., Rebay I. (2005). Post-translational modifications influence transcription factor activity: A view from the ETS superfamily. Bioessays.

[41] Keverne E.B., Pfaff D.W., Tabansky I. (2015). Epigenetic changes in the developing brain: Effects on behavior. Proc. Natl. Acad. Sci. USA.

[42] Wang Y., Hu J., Wu S., Fleishman J.S., Li Y., Xu Y., Zou W., Wang J., Feng Y., Chen J. (2023). Targeting epigenetic and posttranslational modifications regulating ferroptosis for the treatment of diseases. Signal Transduct. Target. Ther..

[43] Santos A.L., Lindner A.B. (2017). Protein Posttranslational Modifications: Roles in Aging and Age-Related Disease. Oxid. Med. Cell Longev..

[44] Creecy A., Brown K.L., Rose K.L., Voziyan P., Nyman J.S. (2021). Post-translational modifications in collagen type I of bone in a mouse model of aging. Bone.

[45] Philpott J.M., Freeberg A.M., Park J., Lee K., Ricci C.G., Hunt S.R., Narasimamurthy R., Segal D.H., Robles R., Cai Y. (2023). PERIOD phosphorylation leads to feedback inhibition of CK1 activity to control circadian period. Mol. Cell.

[46] Nakahata Y., Kaluzova M., Grimaldi B., Sahar S., Hirayama J., Chen D., Guarente L.P., Sassone-Corsi P. (2008). The NAD+-dependent deacetylase SIRT1 modulates CLOCK-mediated chromatin remodeling and circadian control. Cell.

[47] Yuan G., Xu L., Cai T., Hua B., Sun N., Yan Z., Lu C., Qian R. (2019). Clock mutant promotes osteoarthritis by inhibiting the acetylation of NFkappaB. Osteoarthr. Cartil..

[48] Abdalla O., Mascarenhas B., Cheng H.M. (2022). Death of a Protein: The Role of E3 Ubiquitin Ligases in Circadian Rhythms of Mice and Flies. Int. J. Mol. Sci..

[49] Liu H., Craig S.E.L., Molchanov V., Floramo J.S., Zhao Y., Yang T. (2022). SUMOylation in Skeletal Development, Homeostasis, and Disease. Cells.

[50] Katagiri T., Takahashi N. (2002). Regulatory mechanisms of osteoblast and osteoclast differentiation. Oral. Dis..

[51] Nishi H., Hashimoto K., Panchenko A.R. (2011). Phosphorylation in protein-protein binding: Effect on stability and function. Structure.

[52] Marzoll D., Serrano F.E., Shostak A., Schunke C., Diernfellner A.C.R., Brunner M. (2022). Casein kinase 1 and disordered clock proteins form functionally equivalent, phospho-based circadian modules in fungi and mammals. Proc. Natl. Acad. Sci. USA.

[53] Etchegaray J.P., Machida K.K., Noton E., Constance C.M., Dallmann R., Di Napoli M.N., DeBruyne J.P., Lambert C.M., Yu E.A., Reppert S.M. (2009). Casein kinase 1 delta regulates the pace of the mammalian circadian clock. Mol. Cell Biol..

[54] Takahashi A., Mulati M., Saito M., Numata H., Kobayashi Y., Ochi H., Sato S., Kaldis P., Okawa A., Inose H. (2018). Loss of cyclin-dependent kinase 1 impairs bone formation, but does not affect the bone-anabolic effects of parathyroid hormone. J. Biol. Chem..

[55] Lee Y., Kim E.K. (2013). AMP-activated protein kinase as a key molecular link between metabolism and clockwork. Exp. Mol. Med..

[56] Jordan S.D., Lamia K.A. (2013). AMPK at the crossroads of circadian clocks and metabolism. Mol. Cell Endocrinol..

[57] Jeyabalan J., Shah M., Viollet B., Chenu C. (2012). AMP-activated protein kinase pathway and bone metabolism. J. Endocrinol..

[58] Guo Q., Jin Y., Chen X., Ye X., Shen X., Lin M., Zeng C., Zhou T., Zhang J. (2024). NF-kappaB in biology and targeted therapy: New insights and translational implications. Signal Transduct. Target. Ther..

[59] Feng G., Zhao J., Peng J., Luo B., Zhang J., Chen L., Xu Z. (2022). Circadian clock-A promising scientific target in oral science. Front. Physiol..

[60] Gao W., Li R., Ye M., Zhang L., Zheng J., Yang Y., Wei X., Zhao Q. (2022). The circadian clock has roles in mesenchymal stem cell fate decision. Stem Cell Res. Ther..

[61] Li T., Zhang S., Yang Y., Zhang L., Yuan Y., Zou J. (2022). Co-regulation of circadian clock genes and microRNAs in bone metabolism. J. Zhejiang Univ. Sci. B.

[62] Baron R., Rawadi G. (2007). Targeting the Wnt/beta-catenin pathway to regulate bone formation in the adult skeleton. Endocrinology.

[63] Li H., Meng H., Xu M., Gao X., Sun X., Jin X., Sun H. (2023). BMAL1 regulates osteoblast differentiation through mTOR/GSK3beta/beta-catenin pathway. J. Mol. Endocrinol..

[64] Zheng J., Zhang L., Tan Z., Zhao Q., Wei X., Yang Y., Li R. (2022). Bmal1- and Per2-mediated regulation of the osteogenic differentiation and proliferation of mouse bone marrow mesenchymal stem cells by modulating the Wnt/beta-catenin pathway. Mol. Biol. Rep..

[65] Qian Z., Zhang Y., Kang X., Li H., Zhang Y., Jin X., Gao X., Xu M., Ma Z., Zhao L. (2020). Postnatal Conditional Deletion of Bmal1 in Osteoblasts Enhances Trabecular Bone Formation Via Increased BMP2 Signals. J. Bone Miner. Res..

[66] Huang W., Yang S., Shao J., Li Y.P. (2007). Signaling and transcriptional regulation in osteoblast commitment and differentiation. Front. Biosci..

[67] Reale M.E., Webb I.C., Wang X., Baltazar R.M., Coolen L.M., Lehman M.N. (2013). The transcription factor Runx2 is under circadian control in the suprachiasmatic nucleus and functions in the control of rhythmic behavior. PLoS ONE.

[68] Xia C., Tao Y., Li M., Che T., Qu J. (2020). Protein acetylation and deacetylation: An important regulatory modification in gene transcription (Review). Exp. Ther. Med..

[69] Qin Y., Chen Z.H., Wu J.J., Zhang Z.Y., Yuan Z.D., Guo D.Y., Chen M.N., Li X., Yuan F.L. (2023). Circadian clock genes as promising therapeutic targets for bone loss. Biomed. Pharmacother..

[70] Tian Q., Gao S., Zhou X., Zheng L., Zhou Y. (2021). Histone Acetylation in the Epigenetic Regulation of Bone Metabolism and Related Diseases. Stem Cells Int..

[71] Doi M., Hirayama J., Sassone-Corsi P. (2006). Circadian regulator CLOCK is a histone acetyltransferase. Cell.

[72] Chan H.M., La Thangue N.B. (2001). p300/CBP proteins: HATs for transcriptional bridges and scaffolds. J. Cell Sci..

[73] Krishnan R.H., Sadu L., Das U.R., Satishkumar S., Pranav Adithya S., Saranya I., Akshaya R.L., Selvamurugan N. (2022). Role of p300, a histone acetyltransferase enzyme, in osteoblast differentiation. Differentiation.

[74] Bao J., Sack M.N. (2010). Protein deacetylation by sirtuins: Delineating a post-translational regulatory program responsive to nutrient and redox stressors. Cell. Mol. Life Sci..

[75] Asher G., Gatfield D., Stratmann M., Reinke H., Dibner C., Kreppel F., Mostoslavsky R., Alt F.W., Schibler U. (2008). SIRT1 regulates circadian clock gene expression through PER2 deacetylation. Cell.

[76] Abe T., Sato T., Yoda T., Hoshi K. (2019). The period circadian clock 2 gene responds to glucocorticoids and regulates osteogenic capacity. Regen. Ther..

[77] Samsa W.E., Vasanji A., Midura R.J., Kondratov R.V. (2016). Deficiency of circadian clock protein BMAL1 in mice results in a low bone mass phenotype. Bone.

[78] Masri S., Zocchi L., Katada S., Mora E., Sassone-Corsi P. (2012). The circadian clock transcriptional complex: Metabolic feedback intersects with epigenetic control. Ann. N. Y. Acad. Sci..

[79] Jung-Hynes B., Ahmad N. (2009). SIRT1 controls circadian clock circuitry and promotes cell survival: A connection with age-related neoplasms. FASEB J..

[80] Chan W.C.W., Tan Z., To M.K.T., Chan D. (2021). Regulation and Role of Transcription Factors in Osteogenesis. Int. J. Mol. Sci..

[81] Goncalves C.F., Meng Q.J. (2019). Timing metabolism in cartilage and bone: Links between circadian clocks and tissue homeostasis. J. Endocrinol..

[82] Song H.R., Noh Y.S. (2012). Rhythmic oscillation of histone acetylation and methylation at the Arabidopsis central clock loci. Mol. Cells.

[83] Francis M., Pandya M., Gopinathan G., Lyu H., Ma W., Foyle D., Nares S., Luan X. (2019). Histone Methylation Mechanisms Modulate the Inflammatory Response of Periodontal Ligament Progenitors. Stem Cells Dev..

[84] Jin X., Sun X., Ma X., Qin Z., Gao X., Kang X., Li H., Sun H. (2024). SIRT1 maintains bone homeostasis by regulating osteoblast glycolysis through GOT1. Cell Mol. Life Sci..

[85] Suresh B., Lee J., Kim K.S., Ramakrishna S. (2016). The Importance of Ubiquitination and Deubiquitination in Cellular Reprogramming. Stem Cells Int..

[86] Crislip G.R., Johnston J.G., Douma L.G., Costello H.M., Juffre A., Boyd K., Li W., Maugans C.C., Gutierrez-Monreal M., Esser K.A. (2021). Circadian Rhythm Effects on the Molecular Regulation of Physiological Systems. Compr. Physiol..

[87] Ukita Y., Okumura M., Chihara T. (2022). Ubiquitin proteasome system in circadian rhythm and sleep homeostasis: Lessons from Drosophila. Genes. Cells.

[88] Yoo S.H., Mohawk J.A., Siepka S.M., Shan Y., Huh S.K., Hong H.K., Kornblum I., Kumar V., Koike N., Xu M. (2013). Competing E3 ubiquitin ligases govern circadian periodicity by degradation of CRY in nucleus and cytoplasm. Cell.

[89] Reischl S., Vanselow K., Westermark P.O., Thierfelder N., Maier B., Herzel H., Kramer A. (2007). Beta-TrCP1-mediated degradation of PERIOD2 is essential for circadian dynamics. J. Biol. Rhythms.

[90] Xing W., Busino L., Hinds T.R., Marionni S.T., Saifee N.H., Bush M.F., Pagano M., Zheng N. (2013). SCF(FBXL3) ubiquitin ligase targets cryptochromes at their cofactor pocket. Nature.

[91] Maronde E., Schilling A.F., Seitz S., Schinke T., Schmutz I., van der Horst G., Amling M., Albrecht U. (2010). The clock genes Period 2 and Cryptochrome 2 differentially balance bone formation. PLoS ONE.

[92] Srikanta S.B., Cermakian N. (2021). To Ub or not to Ub: Regulation of circadian clocks by ubiquitination and deubiquitination. J. Neurochem..

[93] Jensen E.D., Gopalakrishnan R., Westendorf J.J. (2010). Regulation of gene expression in osteoblasts. Biofactors.

[94] Kim H.J., Kim W.J., Ryoo H.M. (2020). Post-Translational Regulations of Transcriptional Activity of RUNX2. Mol. Cells.

[95] Zhao M., Qiao M., Oyajobi B.O., Mundy G.R., Chen D. (2003). E3 ubiquitin ligase Smurf1 mediates core-binding factor alpha1/Runx2 degradation and plays a specific role in osteoblast differentiation. J. Biol. Chem..

[96] Shimazu J., Wei J., Karsenty G. (2016). Smurf1 Inhibits Osteoblast Differentiation, Bone Formation, and Glucose Homeostasis through Serine 148. Cell Rep..

[97] Itzstein C., Coxon F.P., Rogers M.J. (2011). The regulation of osteoclast function and bone resorption by small GTPases. Small GTPases.

[98] Gohda J., Akiyama T., Koga T., Takayanagi H., Tanaka S., Inoue J. (2005). RANK-mediated amplification of TRAF6 signaling leads to NFATc1 induction during osteoclastogenesis. EMBO J..

[99] Harhaj E.W., Dixit V.M. (2012). Regulation of NF-kappaB by deubiquitinases. Immunol. Rev..

[100] Zhang H.R., Wang Y.H., Xiao Z.P., Yang G., Xu Y.R., Huang Z.T., Wang W.Z., He F. (2024). E3 ubiquitin ligases: Key regulators of osteogenesis and potential therapeutic targets for bone disorders. Front. Cell Dev. Biol..

[101] Soares A.G., Aoki M.S., Miyabara E.H., Deluca C.V., Ono H.Y., Gomes M.D., Moriscot A.S. (2007). Ubiquitin-ligase and deubiquitinating gene expression in stretched rat skeletal muscle. Muscle Nerve.

[102] Tian Y., Ming J. (2022). The role of circadian rhythm in osteoporosis; a review. Front. Cell Dev. Biol..

[103] Severe N., Dieudonne F.X., Marie P.J. (2013). E3 ubiquitin ligase-mediated regulation of bone formation and tumorigenesis. Cell Death Dis..

[104] Lee Y., Chun S.K., Kim K. (2015). Sumoylation controls CLOCK-BMAL1-mediated clock resetting via CBP recruitment in nuclear transcriptional foci. Biochim. Biophys. Acta.

[105] Lyst M.J., Stancheva I. (2007). A role for SUMO modification in transcriptional repression and activation. Biochem. Soc. Trans..

[106] Ma X.N., Li M.Y., Qi G.Q., Wei L.N., Zhang D.K. (2024). SUMOylation at the crossroads of gut health: Insights into physiology and pathology. Cell Commun. Signal.

[107] Lee J., Lee Y., Lee M.J., Park E., Kang S.H., Chung C.H., Lee K.H., Kim K. (2008). Dual modification of BMAL1 by SUMO2/3 and ubiquitin promotes circadian activation of the CLOCK/BMAL1 complex. Mol. Cell Biol..

[108] Cardone L., Hirayama J., Giordano F., Tamaru T., Palvimo J.J., Sassone-Corsi P. (2005). Circadian clock control by SUMOylation of BMAL1. Science.

[109] Kim J.H., Jang J.W., Lee Y.S., Lee J.W., Chi X.Z., Li Y.H., Kim M.K., Kim D.M., Choi B.S., Kim J. (2014). RUNX family members are covalently modified and regulated by PIAS1-mediated sumoylation. Oncogenesis.

[110] Hosoya A., Yukita A., Ninomiya T., Hiraga T., Yoshiba K., Yoshiba N., Kasahara E., Nakamura H. (2013). Localization of SUMOylation factors and Osterix in odontoblast lineage cells during dentin formation and regeneration. Histochem. Cell Biol..

[111] Nayak A., Glockner-Pagel J., Vaeth M., Schumann J.E., Buttmann M., Bopp T., Schmitt E., Serfling E., Berberich-Siebelt F. (2009). Sumoylation of the transcription factor NFATc1 leads to its subnuclear relocalization and interleukin-2 repression by histone deacetylase. J. Biol. Chem..

[112] Kim J.H., Kim N. (2014). Regulation of NFATc1 in Osteoclast Differentiation. J. Bone Metab..

[113] Wang L., Wang P., Xu S., Li Z., Duan D.D., Ye J., Li J., Ding Y., Zhang W., Lu J. (2022). The cross-talk between PARylation and SUMOylation in C/EBPbeta at K134 site participates in pathological cardiac hypertrophy. Int. J. Biol. Sci..

[114] Ren Q., Liu Z., Wu L., Yin G., Xie X., Kong W., Zhou J., Liu S. (2023). C/EBPbeta: The structure, regulation, and its roles in inflammation-related diseases. Biomed. Pharmacother..

[115] Khan S., Duan P., Yao L., Hou H. (2018). Shiftwork-Mediated Disruptions of Circadian Rhythms and Sleep Homeostasis Cause Serious Health Problems. Int. J. Genomics.

[116] Okamoto-Uchida Y., Izawa J., Nishimura A., Hattori A., Suzuki N., Hirayama J. (2019). Post-translational Modifications are Required for Circadian Clock Regulation in Vertebrates. Curr. Genomics.

[117] Garrigue-Antar L., Hartigan N., Kadler K.E. (2002). Post-translational modification of bone morphogenetic protein-1 is required for secretion and stability of the protein. J. Biol. Chem..

[118] Unnanuntana A., Rebolledo B.J., Khair M.M., DiCarlo E.F., Lane J.M. (2011). Diseases affecting bone quality: Beyond osteoporosis. Clin. Orthop. Relat. Res..

[119] Lamande S.R., Bateman J.F. (2020). Genetic Disorders of the Extracellular Matrix. Anat. Rec..

[120] Alcorta-Sevillano N., Infante A., Macias I., Rodriguez C.I. (2022). Murine Animal Models in Osteogenesis Imperfecta: The Quest for Improving the Quality of Life. Int. J. Mol. Sci..

[121] Dudek M., Swift J., Meng Q.J. (2023). The circadian clock and extracellular matrix homeostasis in aging and age-related diseases. Am. J. Physiol. Cell Physiol..

[122] Matsushita T., Wilcox W.R., Chan Y.Y., Kawanami A., Bukulmez H., Balmes G., Krejci P., Mekikian P.B., Otani K., Yamaura I. (2009). FGFR3 promotes synchondrosis closure and fusion of ossification centers through the MAPK pathway. Hum. Mol. Genet..

[123] Besharse J.C. (2001). Coupling an activated MAP kinase to circadian clock output. Neuron.

[124] Song C., Wang J., Kim B., Lu C., Zhang Z., Liu H., Kang H., Sun Y., Guan H., Fang Z. (2018). Insights into the Role of Circadian Rhythms in Bone Metabolism: A Promising Intervention Target?. Biomed. Res. Int..

[125] Spengler M.L., Kuropatwinski K.K., Comas M., Gasparian A.V., Fedtsova N., Gleiberman A.S., Gitlin I.I., Artemicheva N.M., Deluca K.A., Gudkov A.V. (2012). Core circadian protein CLOCK is a positive regulator of NF-kappaB-mediated transcription. Proc. Natl. Acad. Sci. USA.

[126] Kim J.M., Lin C., Stavre Z., Greenblatt M.B., Shim J.H. (2020). Osteoblast-Osteoclast Communication and Bone Homeostasis. Cells.

[127] Juliana N., Azmi L., Effendy N.M., Mohd Fahmi Teng N.I., Abu I.F., Abu Bakar N.N., Azmani S., Yazit N.A.A., Kadiman S., Das S. (2023). Effect of Circadian Rhythm Disturbance on the Human Musculoskeletal System and the Importance of Nutritional Strategies. Nutrients.

[128] Berry J.L., Davies M., Mee A.P. (2002). Vitamin D metabolism, rickets, and osteomalacia. Semin. Musculoskelet. Radiol..

[129] Roodman G.D., Windle J.J. (2005). Paget disease of bone. J. Clin. Investig..

[130] Orsini F., Crotti C., Cincinelli G., Di Taranto R., Amati A., Ferrito M., Varenna M., Caporali R. (2023). Bone Involvement in Rheumatoid Arthritis and Spondyloartritis: An Updated Review. Biology.

[131] Wang X.L., Li L. (2021). Circadian Clock Regulates Inflammation and the Development of Neurodegeneration. Front. Cell Infect. Microbiol..

[132] Hirano A., Fu Y.H., Ptacek L.J. (2016). The intricate dance of post-translational modifications in the rhythm of life. Nat. Struct. Mol. Biol..

[133] Zhou Y., Nakajima R., Shirasawa M., Fikriyanti M., Zhao L., Iwanaga R., Bradford A.P., Kurayoshi K., Araki K., Ohtani K. (2023). Expanding Roles of the E2F-RB-p53 Pathway in Tumor Suppression. Biology.

[134] El-Tanani M., Rabbani S.A., Ali A.A., Alfaouri I.G.A., Al Nsairat H., Al-Ani I.H., Aljabali A.A., Rizzo M., Patoulias D., Khan M.A. (2024). Circadian rhythms and cancer: Implications for timing in therapy. Discov. Oncol..

[135] Chiang C.K., Xu B., Mehta N., Mayne J., Sun W.Y., Cheng K., Ning Z., Dong J., Zou H., Cheng H.M. (2017). Phosphoproteome Profiling Reveals Circadian Clock Regulation of Posttranslational Modifications in the Murine Hippocampus. Front. Neurol..

[136] Wu X., Yang X., Dai Y., Zhao Z., Zhu J., Guo H., Yang R. (2024). Single-cell sequencing to multi-omics: Technologies and applications. Biomark. Res..

[137] Fadul S.M., Arshad A., Mehmood R. (2023). CRISPR-based epigenome editing: Mechanisms and applications. Epigenomics.

[138] Aye-Han N.N., Ni Q., Zhang J. (2009). Fluorescent biosensors for real-time tracking of post-translational modification dynamics. Curr. Opin. Chem. Biol..

[139] Burt L.A., Billington E.O., Rose M.S., Raymond D.A., Hanley D.A., Boyd S.K. (2019). Effect of High-Dose Vitamin D Supplementation on Volumetric Bone Density and Bone Strength: A Randomized Clinical Trial. J. Am. Med. Assoc..

[140] Krzyszczyk P., Acevedo A., Davidoff E.J., Timmins L.M., Marrero-Berrios I., Patel M., White C., Lowe C., Sherba J.J., Hartmanshenn C. (2018). The growing role of precision and personalized medicine for cancer treatment. Technol. Singap. World Sci..

